# Role of Neutrophils in Psoriasis

**DOI:** 10.1155/2020/3709749

**Published:** 2020-06-05

**Authors:** Wen-Ming Wang, Hong-Zhong Jin

**Affiliations:** Department of Dermatology, Peking Union Medical College Hospital, Chinese Academy of Medical Sciences and Peking Union Medical College, Beijing, China

## Abstract

Psoriasis is a common inflammatory disease that can involve the skin, joints, or both. The abnormalities of innate immunity play crucial roles in the pathogenesis of psoriasis. Neutrophils are the most abundant leukocytes in the circulation. Emerging evidences have demonstrated that neutrophils may play a role in autoimmune diseases. The neutrophil-to-lymphocyte ratio (NLR), the activity of neutrophils, and the number of NETotic cells were significantly higher in psoriasis patients compared to healthy controls. The number of low-density granulocytes (LDGs) in the blood of psoriasis patients was significantly higher than those in the control blood. Furthermore, neutrophils may play important roles in the cardiovascular risk in psoriasis. However, the exact role of neutrophils in psoriasis remains unclear. In this review, we highlight the role of neutrophils in the pathogenesis of psoriasis.

## 1. Introduction

**Figure 1:**
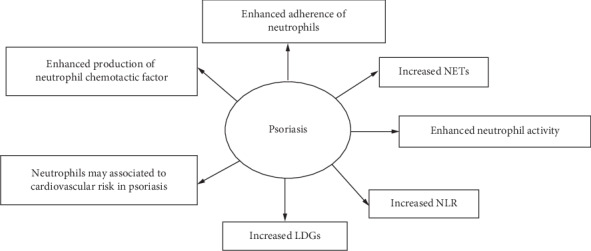
Overview of the role of neutrophils in psoriasis. NETs: neutrophil extracellular traps; NLR: the neutrophil-to-lymphocyte ratio; LDGs: low-density granulocytes.

Psoriasis is a chronic, immune-mediated disorder. The development of psoriasis is attributed to disruptions of the epithelial or immunologic systems, triggered by genetic and environmental factors. [1] The pathogenesis of psoriasis is multifactorial and remains incompletely elucidated. Recent studies have indicated that patients with psoriasis have profound innate immunity disruption, which may play a crucial part in the pathogenesis of the disease. [2, 3] Functional abnormalities in keratinocytes, dendritic cells (DCs), and other components of the innate immune system have been identified in patients with psoriasis. [3] Neutrophils are crucial responders of the innate immune response, but the role played by neutrophils in psoriasis has not been well characterized. This review will discuss normal neutrophil biology and the current understanding of the role and potential mechanisms of neutrophils in psoriasis (Figure 1).

## 2. The Function of Neutrophils

Neutrophils are the most abundant leukocytes in the circulation and the first cells recruited to sites of infection or inflammation. Neutrophils in the circulation are considered to be short-lived cells that undergo constitutive apoptosis after only 24 hours. [4] The migration of neutrophils from the circulation into tissues is a multistep process that involves rolling along the vascular endothelium, adhesion to endothelial cells, extravasation through vascular endothelium, and migration towards inflammatory foci. [4, 5] Neutrophilic clearance of microbes occurs by several processes including phagocytosis, degranulation responses, generation of reactive oxygen species (ROS), and the formation of neutrophil extracellular traps (NETs). [6]

Granules are essential for neutrophils to fulfill their role in innate immunity. After activation of neutrophils, granules can release their contents into the immediate microenvironment. [7] There are three types of granules in neutrophils, as follows: (1) azurophilic granules are reservoirs of antimicrobial compounds, including myeloperoxidase (MPO), defensins, lysozyme, bactericidal/permeability-increasing protein, neutrophil elastase (NE), and cathepsin G [8]; (2) secondary granules are characterized by glycoprotein lactoferrin, including NGAL and hCAP-18 [9]; and (3) gelatinase granules, which are considered to be the site of storage of metalloproteases such as gelatinase and leukolysin. [10] In response to infections, neutrophils can lead to the destruction of pathogens through the release of reactive oxygen species (ROS; by MPO and NADPH oxidase activity) and reactive nitrogen species (RNS; by nitric oxide synthase (NOS)). [11, 12] Despite their beneficial role against pathogens, chronic or uncontrolled ROS production can contribute to lipid membrane damage, DNA damage, and genetic instability. [11, 13] Neutrophil extracellular traps (NETs), which are generated by activated neutrophils, play crucial roles in the innate immune system. [14] NETs are composed of cell-free DNA, histones, antimicrobial proteins, danger molecules, and autoantigens [14, 15] and play vital roles in the control of bacterial, viral, fungal, and parasitic infections. [14, 16] Previous studies have demonstrated that neutrophils contribute to the onset of several autoimmune and inflammatory diseases, such as systemic lupus erythematosus (SLE), [17] rheumatoid arthritis (RA), [4] inflammatory bowel diseases, atherosclerosis, and others. [12]

## 3. The Function of Neutrophils in Psoriasis

CD62L, CD11b, and CD66b can be used as markers of neutrophil activity. CD62L can be cleaved from activated neutrophils. The expression of CD62L on neutrophils from severely psoriatic patients was lower than those from moderately psoriatic individuals and normal healthy controls, whereas neutrophils from psoriasis patients who received biologic treatments (infliximab or ustekinumab) had normal CD62L levels. Expression of CD11b and CD66b was increased in activated neutrophils. It has been shown that CD11b and CD66b were higher in severe psoriasis patients than moderately psoriatic patients and normal healthy controls, while neutrophils from psoriasis patients receiving biologic treatment (infliximab or ustekinumab) had normal CD11b and CD66b levels. [18] This study indicated that there might be increased neutrophil activity in psoriasis. Thy-1 is an adhesion molecule and is strongly expressed on endothelial cells (ECs) in psoriatic lesions. Compared with polymorphonuclear cells from healthy controls, polymorphonuclear cells derived from psoriasis patients have a stronger ability to adhere to Thy-1 and can be decreased by standard psoriasis treatment. [19] However, the exact role played by aberrant neutrophil function in the pathogenesis of psoriasis remains unclear.

## 4. Neutrophilic Leukocytosis in Psoriasis

Psoriasis is an immune-mediated disease, which is characterized by local and systemic inflammation. [20] The infiltration of neutrophils into the skin can be induced by several chemotactic factors, such as IL-8, NAP-2, NAP-3, and LPS. [21] In psoriatic lesions, neutrophils infiltrate into the dermis and epidermis and can form Kogoj or Munro's microabscesses. [22] Previous studies showed that IL-8 was expressed in the majority of neutrophils in psoriatic lesions. [23]

Flaky skin mice (*fsn*/*fsn*) show neutrophil-derived microabscess formation in the epidermis and hyperproliferative inflammatory skin. [24] Depletion of neutrophils by RB6-8C5 monoclonal antibody can significantly reduce the epidermal thickness. Epidermal thickness can also be reduced by the blocking of integrin *α*M*β*2 (CD11b/CD18), which mediates neutrophil localization through binding to the intercellular adhesion molecule-1. [25] Leukotriene B4 (LTB4), which was found to be upregulated in psoriatic lesions, is a chemoattractant for neutrophils and can induce the influx of polymorphonuclear cells into the epidermis and dermis. [26] In imiquimod-induced psoriasis mouse model, depletion of neutrophils with anti-Ly-6G antibody can ameliorate the severity of psoriasis. Moreover, CXCR2 and LTB4 receptor 1 can promote neutrophil recruitment into psoriatic skin cooperatively. [27]

The neutrophil-to-lymphocyte ratio (NLR) has been shown to be useful in the assessment of clinical severity and outcomes in several chronic inflammatory diseases. [20, 28, 29] A meta-analysis conducted by Paliogiannis and colleagues, which include 1067 psoriasis patients and 799 healthy controls, showed that the NLR was significantly higher in patients with psoriasis (standardized mean difference = 0.69, 95% CI 0.53–1.85, *p* < 0.001). However, there were no significant differences in NLR values according to the severity of disease (PASI < 10 or PASI > 10) [20]. After 12 weeks, there was a significant reduction in the NLR of antibody-treated (brodalumab and ustekinumab) psoriasis patients, compared with the NLR of placebo patients. [30] A study performed by Asahina et al. demonstrated that in psoriasis vulgaris patients, the NLR-high subgroups exhibited significantly higher PASI scores compared with the NLR-low subgroups, and NLR also increased with increasing PASI scores. In addition, after treatment with biologics (infliximab, adalimumab, and ustekinumab) for 12 months, patients showed a decreased NLR in parallel with a decrease in C-reactive protein (CRP) [31]. Polat and colleagues conducted a study on 46 chronic plaque psoriasis patients and 46 healthy controls from the Turkish population, showing that NLR was significantly higher in chronic plaque psoriasis patients compared with healthy controls. Moreover, PASI score was positively correlated with NLR. [32] Taking these findings together, studies have consistently shown increased NLR in psoriasis patients, with the decrease in NLR after treatment representing not only a treatment effect but also a reflection of disease activity. Thus, future investigation of the relationship between NLR and the severity of psoriasis is warranted.

## 5. Low-Density Granulocytes and Psoriasis

Low-density granulocytes (LDGs) are a subset of neutrophils and can be purified from the less dense peripheral blood mononuclear cell (PBMC) fraction. LDGs have a strong ability to generate NETs. [33] Previous studies have revealed that the number of LDGs in the blood of psoriasis patients was significantly higher than in the control blood. In addition, under conditions without any stimulation, the ability of LDGs to form NETs was stronger than the control or psoriatic neutrophils [34]. In a study of LDGs derived from SLE, LDGs have similar cell surface markers but different nuclear morphology compared with mature autologous or healthy control neutrophils. LDGs also have an enhanced ability to secrete TNF and type I and type II IFNs. [17, 35] However, the role of LDGs in the pathogenesis of psoriasis requires further study to become fully understood.

## 6. NETs and Psoriasis

NETosis refers to the release of NETs and can be divided into three models. First, *suicidal NETosis*, which can last for 2–4 h. After being triggered by stimuli, ROS can be induced through the MEK-extracellular-signal-regulated kinase (ERK) signaling pathway, leading to the activation of peptidyl arginase deaminase 4 (PAD4), which is a nuclear enzyme allowing the conversion of arginine to citrulline on histones and decondensation of chromatin. [36] The second model of NETosis is triggered by stimuli that can recognize toll-like receptors (TLRs) and the complement receptor for C3 protein. Moreover, it is not dependent on ROS and the MEK/ERK signaling pathway. The third model of NETosis is dependent on mitochondrial ROS production, and mitochondrial DNA is released during the process [37]. NETs might play a role in several diseases, such as SLE, RA, atherosclerosis, and cancer [36], but little is known about the role of NETs in the pathogenesis of psoriasis.

LL-37 is an antimicrobial component of NETs, which can also form complexes with DNA or RNA. Keratinocytes of psoriatic lesions showed a higher level of LL-37 [3]. Plasmacytoid DCs (pDCs) can be activated by LL-37/DNA complexes and produce type I interferons, while myeloid DCs can be activated by LL-37/RNA complexes and produce TNF-*α* and IL-6 [38, 39]. Neutrophil elastase (NE) and secretory leukocyte proteinase inhibitor (SLPI), which can promote the secretion of type I interferons by pDCs, can be found in NETs. Furthermore, SLPI+ neutrophils and NETs can be found colocalized with pDCs in psoriatic skin [40].

Using fluorescent microscopy, Hu et al. showed that there were higher numbers of NETotic cells in the peripheral blood of psoriasis patients than in healthy controls and patients with eczema. The number of NETs in the peripheral blood of psoriatic patients was also correlated with disease severity. In addition, NETs were observed in the majority (18/20) of psoriasis skin specimens, while NETs were not seen in lesions of patients with eczema (0/20). Using immunohistochemical analysis, *β-*defensin-2 (HBD-2) was found to be higher in psoriasis lesional skin than normal skin and eczema lesional skin. Furthermore, NETs can induce the expression of HBD-2 mRNA and protein in keratinocytes [41]. The findings indicate that there was a higher number of NETs in psoriasis patients and that NETs may correlate with disease severity in these patients. Future studies should confirm these findings, then assess the exact role that NETs play in the initiation and maintenance of psoriasis.

## 7. Association between Neutrophils and Cardiovascular Risk in Psoriasis

Epidemiological studies have shown that psoriasis is a risk factor for major adverse cardiovascular events and cardiovascular mortality [42]. Common inflammatory pathways may contribute to this interaction, and this hypothesis is confirmed by the finding that improvement in disease severity of psoriasis was associated with reduced aortic vascular inflammation and coronary plaque burden [43]. However, the exact mechanisms are still unknown [42, 44].

Previous studies have reported that pulse wave velocity (PWV), augmentation index (AIx), NLR, and heart rate were higher in psoriasis patients compared with healthy controls. In addition, after stratification by heart rate, PWV and AIx were significantly associated with psoriasis. The study also indicated that NLR may be related to vascular dysfunction in psoriasis patients [45]. Consistent with this, Yurtdas et al. have demonstrated that psoriasis patients had higher NLR and high-sensitivity CRP compared with controls. Lower aortic velocity propagation (AVP) and higher carotid intima-media thickness (CIMT) values were found in psoriasis patients. In addition, lower AVP and higher CIMT values can be predicted by NLR. This study is a reminder that NLR may be a predictor of subclinical atherosclerosis in patients with psoriasis [46].

Arterial inflammation, which is measured using average aortic target-to-background ratio (TBR) using FDG PET/CT, was increased in psoriatic patients compared with controls, and there was also a positive association between PASI score and aortic TBR. Additionally, the levels of S100A8/A9 and neutrophil elastase-1 were elevated in the serum of psoriasis patients. CD16 and CD62L, which can be cleaved from activated leukocytes, were decreased on the surface of neutrophils from psoriasis patients. This study suggested that neutrophils may be activated in psoriasis [47].

The KC-Tie2 mouse is a keratinocyte-specific, Tie2-overexpressing psoriasis model with cutaneous expression of IL-23 and IL-17A. After treatment with antibodies targeting IL-23, IL-17A, or IL-17RA, the KC-Tie2 mouse showed a significant improvement in skin inflammation and lengthened occlusive thrombus formation time. The alleviation of skin inflammation paralleled decreasing numbers of splenic neutrophils (CD11b+, Ly6G+) [48].

## 8. Neutrophils and IL-17A

Neutrophils are the major source of IL-17A in psoriatic skin lesions [49]. IL-17 can promote the expression of HBD-2, S100A7, S100A8, S100A9, and LL37 by keratinocytes. Secukinumab targets IL-17A. In psoriasis patients, clinical responses to secukinumab were associated with clearance of cutaneous neutrophils and reduction of IL-17-inducible neutrophil chemoattractants derived from keratinocytes, such as CXCL1 and CXCL8 [50]. It was suggested that there may be crosstalk between neutrophils and keratinocytes which involved neutrophil-derived IL-17 in psoriasis. IL-17E in lesional psoriatic skin was produced by keratinocytes and correlated with the number of neutrophils [51]. In K14-IL-17A^ind/+^ mice, blocking of IL-6 reduced IL-17A-induced neutrophil microabscess formation in the epidermis [52]. Psoriatic keratinocytes were more efficient than healthy keratinocytes in increasing the lifespan of neutrophils and promoting the production of superoxidation of neutrophils [53].

Using K14-IL-17A^ind/+^ mice, Karbach and colleagues showed that impaired vascular function, increased ROS formation, endothelial dysfunction, and arterial hypertension driven by MPO+ cells can be induced by the overexpression of IL-17A in keratinocytes. In addition, blocking of TNF-*α* or IL-6 can attenuate skin manifestation and vascular phenotype of K14-IL-17A^ind/+^ mice [54].

K14-IL-17A^ind/+^ mice can suffer from an early-onset severe psoriasis-like phenotype, while homozygous CD11c-IL-17A^ind/ind^ and heterozygous CD11c-IL-17A^ind/+^ mice demonstrate delayed onset of moderate psoriasis-like skin disease. Blocking of IL-17A cannot improve skin and vascular disease in K14-IL-17A^ind/+^ mice but can improve skin lesions and vascular dysfunction in CD11c-IL-17A^ind/ind^ mice. Imiquimod-induced psoriasis-like skin inflammation can be significantly attenuated by anti-IL-17A treatment, whereas this psoriasis model has no vascular dysfunction. Blocking of IL-17A can downregulate oxidative stress levels, proinflammatory cytokines, and vascular inflammation [55]. This study indicated that IL-17A might be a link between vascular disease and psoriasis. In conclusion, neutrophils may play a role in the cardiovascular risk of psoriasis patients, but more studies are needed to fully elucidate the mechanism.

## 9. Conclusions

Neutrophils play a crucial role in the development of psoriasis. Aberrant function, phenotype, and number of neutrophils are found in psoriatic patients. NETosis may contribute to the dysfunction of the innate immunity in psoriasis patients and warrants further investigation. The current knowledge of LDGs in psoriasis is limited, but worthy of study. IL-17 is an important cytokine in the pathogenesis of psoriasis and may have a negative effect on cardiovascular risk and vascular function in psoriatic patients. Further studies to better elucidate the role of neutrophils and the underlying mechanisms are therefore necessary.

## References

[1] von Csiky-Sessoms S., Lebwohl M. (2019). What’s New in Psoriasis. *Dermatologic Clinics*.

[2] Liang Y., Sarkar M. K., Tsoi L. C., Gudjonsson J. E. (2017). Psoriasis: a mixed autoimmune and autoinflammatory disease. *Current Opinion in Immunology*.

[3] Nestle F. O., Kaplan D. H., Barker J. (2009). Psoriasis. *New England Journal of Medicine*.

[4] Wright H. L., Moots R. J., Edwards S. W. (2014). The multifactorial role of neutrophils in rheumatoid arthritis. *Nature Reviews Rheumatology*.

[5] Henson P. M. (1972). Pathologic mechanisms in neutrophil-mediated injury. *The American Journal of Pathology*.

[6] Jones H. R., Robb C. T., Perretti M., Rossi A. G. (2016). The role of neutrophils in inflammation resolution. *Seminars in Immunology*.

[7] Borregaard N., Cowland J. B. (1997). Granules of the human neutrophilic polymorphonuclear leukocyte. *Blood*.

[8] Faurschou M., Borregaard N. (2003). Neutrophil granules and secretory vesicles in inflammation. *Microbes and Infection*.

[9] Lacy P. (2005). The role of Rho GTPases and SNAREs in mediator release from granulocytes. *Pharmacology & Therapeutics*.

[10] Amulic B., Cazalet C., Hayes G. L., Metzler K. D., Zychlinsky A. (2012). Neutrophil function: from mechanisms to disease. *Annual Review of Immunology*.

[11] Bonavita O., Massara M., Bonecchi R. (2016). Chemokine regulation of neutrophil function in tumors. *Cytokine & Growth Factor Reviews*.

[12] Elloumi N., Ben Mansour R., Marzouk S. (2017). Differential reactive oxygen species production of neutrophils and their oxidative damage in patients with active and inactive systemic lupus erythematosus. *Immunology Letters*.

[13] Tazawa H., Okada F., Kobayashi T. (2003). Infiltration of neutrophils is required for acquisition of metastatic phenotype of benign murine fibrosarcoma cells: implication of inflammation-associated carcinogenesis and tumor progression. *The American Journal of Pathology*.

[14] van Dam L. S., Rabelink T. J., van Kooten C., Teng Y. K. O. (2019). Clinical implications of excessive neutrophil extracellular trap formation in renal autoimmune diseases. *Kidney International Reports*.

[15] Li R. H. L., Tablin F. (2018). A comparative review of neutrophil extracellular traps in sepsis. *Frontiers in Veterinary Science*.

[16] Brinkmann V., Reichard U., Goosmann C. (2004). Neutrophil extracellular traps kill bacteria. *Science*.

[17] Kaplan M. J. (2011). Neutrophils in the pathogenesis and manifestations of SLE. *Nature Reviews Rheumatology*.

[18] Yamanaka K., Umezawa Y., Yamagiwa A. (2014). Biologic therapy improves psoriasis by decreasing the activity of monocytes and neutrophils. *The Journal of Dermatology*.

[19] Wetzel A., Wetzig T., Haustein U. F. (2006). Increased neutrophil adherence in psoriasis: role of the human endothelial cell receptor Thy-1 (CD90). *The Journal of Investigative Dermatology*.

[20] Paliogiannis P., Satta R., Deligia G. (2019). Associations between the neutrophil-to-lymphocyte and the platelet-to-lymphocyte ratios and the presence and severity of psoriasis: a systematic review and meta-analysis. *Clinical and Experimental Medicine*.

[21] Lorthois I., Asselineau D., Seyler N., Pouliot R. (2017). Contribution of in vivo and organotypic 3D models to understanding the role of macrophages and neutrophils in the pathogenesis of psoriasis. *Mediators of Inflammation*.

[22] Kaneko F., Itoh N., Yoshida H., Suzuki M., Ono I. (1991). The cell-components and cytokines in the subcorneal microabscess of psoriasis. *Fukushima Journal of Medical Science*.

[23] Duan H., Koga T., Kohda F., Hara H., Urabe K., Furue M. (2001). Interleukin-8-positive neutrophils in psoriasis. *Journal of Dermatological Science*.

[24] Sundberg J. P., France M., Boggess D. (1997). Development and progression of psoriasiform dermatitis and systemic lesions in the flaky skin (fsn) mouse mutant. *Pathobiology*.

[25] Schön M., Kubitza R. C., Ruzicka T., Schön M. P., Denzer D. (2000). Critical role of neutrophils for the generation of psoriasiform skin lesions in flaky skin mice. *The Journal of Investigative Dermatology*.

[26] Keijsers R. R. M. C., Hendriks A. G. M., van Erp P. E. J. (2014). In vivo induction of cutaneous inflammation results in the accumulation of extracellular trap-forming neutrophils expressing ROR*γ*t and IL-17. *The Journal of Investigative Dermatology*.

[27] Sumida H., Yanagida K., Kita Y. (2014). Interplay between CXCR2 and BLT1 facilitates neutrophil infiltration and resultant keratinocyte activation in a murine model of imiquimod-induced psoriasis. *Journal of Immunology*.

[28] Paliogiannis P., Fois A. G., Sotgia S. (2018). Neutrophil to lymphocyte ratio and clinical outcomes in COPD: recent evidence and future perspectives. *Eur Respir Rev*.

[29] Erre G. L., Paliogiannis P., Castagna F. (2019). Meta-analysis of neutrophil-to-lymphocyte and platelet-to-lymphocyte ratio in rheumatoid arthritis. *European Journal of Clinical Investigation*.

[30] Zhang L., Wiles C., Martinez L. R., Han G. (2017). Neutrophil-to-lymphocyte ratio decreases after treatment of psoriasis with therapeutic antibodies. *Journal of the European Academy of Dermatology and Venereology*.

[31] Asahina A., Kubo N., Umezawa Y., Honda H., Yanaba K., Nakagawa H. (2017). Neutrophil-lymphocyte ratio, platelet-lymphocyte ratio and mean platelet volume in Japanese patients with psoriasis and psoriatic arthritis: response to therapy with biologics. *The Journal of Dermatology*.

[32] Polat M., Bugdayci G., Kaya H., Oguzman H. (2017). Evaluation of neutrophil-to-lymphocyte ratio and platelet-to-lymphocyte ratio in Turkish patients with chronic plaque psoriasis. *Acta Dermatovenerologica Alpina Pannonica et Adriatica*.

[33] Carmona-Rivera C., Kaplan M. J. (2013). Low-density granulocytes: a distinct class of neutrophils in systemic autoimmunity. *Seminars in Immunopathology*.

[34] Lin A. M., Rubin C. J., Khandpur R. (2011). Mast cells and neutrophils release IL-17 through extracellular trap formation in psoriasis. *Journal of Immunology*.

[35] Denny M. F., Yalavarthi S., Zhao W. (2010). A distinct subset of proinflammatory neutrophils isolated from patients with systemic lupus erythematosus induces vascular damage and synthesizes type I IFNs. *Journal of Immunology*.

[36] Jorch S. K., Kubes P. (2017). An emerging role for neutrophil extracellular traps in noninfectious disease. *Nature Medicine*.

[37] Delgado-Rizo V., Martínez-Guzmán M. A., Iñiguez-Gutierrez L., García-Orozco A., Alvarado-Navarro A., Fafutis-Morris M. (2017). Neutrophil extracellular traps and its implications in inflammation: an overview. *Frontiers in Immunology*.

[38] Pinegin B., Vorobjeva N., Pinegin V. (2015). Neutrophil extracellular traps and their role in the development of chronic inflammation and autoimmunity. *Autoimmunity Reviews*.

[39] Hoffmann J. H. O., Enk A. H. (2016). Neutrophil extracellular traps in dermatology: caught in the NET. *Journal of Dermatological Science*.

[40] Skrzeczynska-Moncznik J., Wlodarczyk A., Zabieglo K. (2012). Secretory leukocyte proteinase inhibitor-competent DNA deposits are potent stimulators of plasmacytoid dendritic cells: implication for psoriasis. *Journal of Immunology*.

[41] Hu S. C.-S., Yu H.-S., Yen F.-L., Lin C.-L., Chen G.-S., Lan C.-C. E. (2016). Neutrophil extracellular trap formation is increased in psoriasis and induces human *β*-defensin-2 production in epidermal keratinocytes. *Scientific Reports*.

[42] Mehta N. N., Yu Y. D., Pinnelas R. (2011). Attributable risk estimate of severe psoriasis on major cardiovascular events. *The American Journal of Medicine*.

[43] Dey A. K., Joshi A. A., Chaturvedi A. (2017). Association between skin and aortic vascular inflammation in patients with psoriasis: a case-cohort study using positron emission tomography/computed tomography. *JAMA Cardiology*.

[44] Davidovici B. B., Sattar N., Jörg P. C. (2010). Psoriasis and systemic inflammatory diseases: potential mechanistic links between skin disease and co-morbid conditions. *The Journal of Investigative Dermatology*.

[45] Sunbul M., Seckin D., Durmus E. (2015). Assessment of arterial stiffness and cardiovascular hemodynamics by oscillometric method in psoriasis patients with normal cardiac functions. *Heart and Vessels*.

[46] Yurtdaş M., Yaylali Y. T., Kaya Y., Özdemir M., Özkan İ., Aladağ N. (2014). Neutrophil-to-lymphocyte ratio may predict subclinical atherosclerosis in patients with psoriasis. *Echocardiography*.

[47] Naik H. B., Natarajan B., Stansky E. (2015). Severity of psoriasis associates with aortic vascular inflammation detected by FDG PET/CT and neutrophil activation in a prospective observational study. *Arteriosclerosis, Thrombosis, and Vascular Biology*.

[48] Li Y., Golden J. B., Camhi M. I. (2018). Protection from psoriasis-related thrombosis after inhibition of IL-23 or IL-17A. *The Journal of Investigative Dermatology*.

[49] Katayama H. (2018). Development of psoriasis by continuous neutrophil infiltration into the epidermis. *Experimental Dermatology*.

[50] Reich K., Papp K. A., Matheson R. T. (2015). Evidence that a neutrophil-keratinocyte crosstalk is an early target of IL-17A inhibition in psoriasis. *Experimental Dermatology*.

[51] Senra L., Stalder R., Alvarez Martinez D., Chizzolini C., Boehncke W. H., Brembilla N. C. (2016). Keratinocyte-derived IL-17E contributes to inflammation in psoriasis. *The Journal of Investigative Dermatology*.

[52] Croxford A. L., Karbach S., Kurschus F. C. (2014). IL-6 regulates neutrophil microabscess formation in IL-17A-driven psoriasiform lesions. *The Journal of Investigative Dermatology*.

[53] Guerard S., Allaeys I., Martin G., Pouliot R., Poubelle P. E. (2013). Psoriatic keratinocytes prime neutrophils for an overproduction of superoxide anions. *Archives of Dermatological Research*.

[54] Karbach S., Croxford A. L., Oelze M. (2014). Interleukin 17 drives vascular inflammation, endothelial dysfunction, and arterial hypertension in psoriasis-like skin disease. *Arteriosclerosis, Thrombosis, and Vascular Biology*.

[55] Schüler R., Brand A., Klebow S. (2019). Antagonization of IL-17A attenuates skin inflammation and vascular dysfunction in mouse models of psoriasis. *The Journal of Investigative Dermatology*.

